# Production performance of Alabio ducks (*Anas platyrhynchos Borneo*) under different levels of drinking water salinity

**DOI:** 10.5455/javar.2022.i589

**Published:** 2022-06-26

**Authors:** Abrani Sulaiman, Surya Rahmatullah, Hefni Effendi, Gamaliel Simanungkalit

**Affiliations:** 1 Department of Animal Science, Faculty of Agriculture, Lambung Mangkurat University, Banjarbaru, Indonesia; 2 Center for Environmental Science PPLH, IPB University, Bogor, Indonesia; 3 School of Environmental and Rural Science, University of New England, Armidale, Australia

**Keywords:** Alabio ducks, water salinity, eggs

## Abstract

**Objective:**

To examine the effects of the salinity level of drinking water on the egg production and quality of Alabio ducks.

**Materials and Methods:**

A total of 60 female Alabio ducks, aged 6 months, were subjected to this study. All ducks were kept in stage-type cages (1 m length × 1 m width × 0.5 m height), where each cage was inhabited by 4 ducks for 56 days of experimentation. All ducks were offered a mixed ration ad libitum for laying ducks, according to the nutritional requirements for egg-type ducks. The treatment in this study was drinking water with five stratified salinity levels, namely P0 = freshwater (0% salinity); P1 = water with a salinity of 0.75 practical salinity unit (PSU) (equal to 0.75 g NaCl/l); P2 = water with a salinity of 1.5 PSU (1.5 gm/l); P3 = water with a salinity of 2.5 PSU (2.5 gm/l); and P4 = water with a salinity of 3 PSU (3.0 gm/l). Observations were made on water intake, feed intake, egg production, and egg quality (egg weight, egg shape index density, shell proportion, shell thickness, yolk index, albumen index, and Haugh unit).

**Results:**

The results showed that the difference in salinity levels in drinking water from 0.75 PSU to 3 PSU did not affect water intake, feed intake, egg production, or egg quality of Alabio ducks for the first 56 days of the laying period (*p* > 0.05).

**Conclusions:**

It was concluded that Alabio ducks have a good tolerance for drinking water salinity of up to 3 PSU, or equal to 3 gm/l NaCl.

## Introduction

Alabio ducks (Anas platyrhynchos Borneo) are a local duck breed in Indonesia and are mainly reared in the province of South Kalimantan [1]. They are considered the native livestock germplasm of Indonesia, with a total population of 4.4 million heads spread across 13 districts in South Kalimantan [2]. These ducks have been identified as having the ability to perform well in harsh environmental conditions, with a limited supply of required nutrients [3]. Although Alabio ducks are a dual-purpose breed, they have the potential as superior laying ducks that plays an essential role in a socioeconomic aspect by providing a livelihood to smallholders and food for humans [4]. As waterfowls, Alabio ducks have a suitable habitat in the aquatic environment, mainly swampy areas, which causes the potential for high egg production and quality to be likely constrained by contaminated drinking water in the changing environment [5]. The water contamination in South Kalimantan mainly derives from seawater intrusion into the river, altering the salinity of the water [6].

Salinity is the level of saline or salt (sodium chloride, NaCl) content dissolved in water. It can also refer to the salt content in the soil. The source of salt dissolved in seawater is the weathering process of rocks and gases that come out of the ocean ridges and underwater volcanoes [7]. The salt content in most lakes, rivers, and natural waterways is low, and so the water in these places is categorized as fresh water because the salt content is less than 0.05 practical salinity unit (PSU), equivalent to 0.05 gm/l NaCl. According to the NaCl content, water salinity can be categorized as 1) brackish (0.05–3 gm/l), 2) saline (3–5 gm/l), and brine (> 5 gm/l) [8]. Chloride (55%), sodium (31%), sulfate (8%), magnesium (4%), calcium (1%), potassium (1%), and others (1% of bicarbonate, bromide, boric acid, strontium, and fluoride each) are the most common salts found in seawater. At the farmer level, a problem arising in the duck farming system in tidal swamp areas and riverbanks of South Kalimantan is the availability of fresh and clean water during the long dry season. According to Hidayat [9], the seawater intrusion in the area changes the river water’s salinity to become brackish and even salty, indicating an increase in water salinity if the seawater experiences a tide that is not balanced by freshwater due to rain in the upstream part of the river. This ongoing situation has been sparking a drastic decrease in the production performance of Alabio ducks. Although Wang et al. [10] stated that Cl was essential in maintaining fluid and electrolyte balance in the kidneys, the recommended amount of Cl in the diet should be maintained at 0.15%.

Research on the effect of the salinity of drinking water given to Alabio ducks is currently unavailable, so the available information is minimal. However, farmers often respond to the salinity problem by intensively caging the ducks or selling the ducks to minimize the production cost. Therefore, this study aims to determine the effect of water salinity on the egg production and quality of Alabio ducks. The results of this study could be used by the farmers rearing the ducks in swamplands and coastal areas regarding the use of saline water for Alabio ducks.

## Materials and Methods

### Ethical approval and experimental site

Procedures and research protocols were approved by the Faculty of Agriculture, Lambung Mangkurat University (#FP-ULM 010718) under the Indonesian Government Regulation No. 95 of the Year 2012 on Veterinary Public Health and Animal Welfare. The experiment was conducted at Tinggiran Luar 2 Village, Barito Kuala Regency, the Province of South Kalimantan, Indonesia (3°18’32.4”S; 114°32’31.5”E; 1.1 m altitude), from 2 July 2018 to 26 August 2018.

### Animals and instrumentation

Sixty female Alabio ducks aged 6 months were subject to this current study. All ducks were kept in stage-type cages (1 m length × 1 m width × 0.5 m height), where each cage was inhabited by four ducks. According to the nutritional requirements for egg-type ducks, all ducks were offered a mixed ration for laying ducks [11]. The major nutrient composition of the ration was as follows: ME = 2,600 kcal/kg; %CP = 17%; methionine = 0.45%; lysine = 0.80%; calcium = 3.5%; and phosphorus = 0.35%. The laboratory analysis for egg quality was undertaken at the Animal Science laboratory of Lambung Mangkurat University. The equipment used in this study was drinking bowls, feed containers, scales, analytical balances, digital calipers, electronic digital micrometers, measuring cups, glasses, egg trays, thermometers, and hygrometers. 

### Experimental procedures

The 60 ducks were arbitrarily divided into 15 groups and allocated to 5treatments of drinking water salinity for 56 days of experimentation. Each treatment had three replicates, with each replicate consisting of four ducks. The treatment groups were as follows: P0 = freshwater (0% salinity); P1 = water with a salinity of 0.75 PSU (equal to 0.75 g NaCl/l); P2 = water with a salinity of 1.5 PSU (1.5 gm/l); P3 = water with a salinity of 2.5 PSU (2.5 gm/l); and P4 = water with a salinity of 3 PSU (3.0 gm/l). The salinity levels were obtained from a mixture of freshwater and seawater sourced from the sea near the estuary of the Barito River, South Kalimantan. The salinity of the seawater being used was 35 PSU. A standard dilution formula for a liquid solution was used to obtain the drinking water with the desired salinity level. The formula was expressed as *M*1 × *V*1 = *M*2 × *V*2, where *M*1 is the salinity of seawater (ppt), *M*2 is the desired salinity (ppt), *V*1 is the volume of seawater required (l), and *V*2 is the volume of drinking water requirement that has been determined (l) [12]. After determining the solution, the drinking water was given ad libitum. 

### Observed variables

The observed variables in this current study were feed consumption (gm/day), drinking water consumption (ml/day), egg production (%), and egg quality. The daily feed and water intakes were measured by subtracting the initial weight of feed and water offered in the morning (07:00 h) with the refusals within 24 h. Egg production was calculated weekly for 8 weeks (56 days), while the egg quality measurements were obtained by taking 2 eggs from each individual per week, yielding a total sample of 240 eggs. The measurements included egg shape index (%), density (gm/ml), shell proportion (%), shell thickness (µm), yolk index (%), albumen index (%), and Haugh unit (HU) [13]. The formulas for these measurements are described as follows:


$$\mathrm{Egg}\;\mathrm{shape}\;\mathrm{index}=\frac{\mathrm{Egg}\;\mathrm{width}\;(\mathrm{mm})}{\mathrm{Egg}\;\mathrm{length}\;(\mathrm{mm})}\times 100\%$$

$$\mathrm{Density}\;=\;\frac{\mathrm{Egg}\;\mathrm{weight}\;(\mathrm{gm})}{\mathrm{Egg}\;\mathrm{volume}\;(\mathrm{ml})}$$

$$\mathrm{Shell}\;\mathrm{proportion}\;=\;\frac{\mathrm{Shell}\;\mathrm{weight}\;(\mathrm{gm})}{\mathrm{Egg}\;\mathrm{weight}\;(\mathrm{gm})}\times 100\%$$

$$\mathrm{Yolk}\;\mathrm{index}\;=\;\frac{\mathrm{Yolk}\;\mathrm{height}\;(\mathrm{mm})}{\mathrm{Yolk}\;\mathrm{diameter}\;(\mathrm{mm})}\times 100\%$$

$$\mathrm{Albumen}\;\mathrm{index}\;=\;\frac{\mathrm{Albumen}\;\mathrm{height}\;(\mathrm{mm})}{\left[\frac{\mathrm{Albumen}\;\mathrm{length}\;(\mathrm{mm})+\mathrm{Albumen}\;\mathrm{width}\;(\mathrm{mm})}{2}\right]}\times 100\%$$

Haugh unit = 100 log_10_ (H - 1.7 W^0.37^ + 7.56)

### Data analysis

Data were statistically analysed using a linear mixed-effects model by means of the *lmerTest* package of R statistics [14]. The statistical model used is as follows:

*Y*_ijk_ *μ* *β*_i_ *β*_j_ *β*_ijk_ *ε*_ijk_

where *Y*_ijk_ is the observed variables, *μ* is the overall mean, *β*0 is the treatment (water salinity) as a fixed effect (*i* = 1–5), *β*1 is the sampling time as a fixed effect (*j* = 1–8), *β*2 is the group as a random effect (*k *= 1–5), and *ε*_ijk_ is the random error.

## Results

### Effects of drinking water salinity on feed and water intake in Alabio ducks

Feed and water intakes of Alabio ducks at the different levels of drinking water salinity are presented in Table 1. Overall, there was no significant influence of drinking water salinity from 0.75 to 3 PSU on the ducks’ water intake (*p* > 0.05). The same result was found for feed intake (*p* > 0.05), which showed that Alabio ducks could handle a salinity level of 3 gm/l NaCl without their ability to eat or drink getting worse. 

### Effects of drinking water salinity on egg production and quality of Alabio ducks

Table 2 describes the egg production and quality of Alabio ducks after being offered water with different salinity levels. Egg production over 56 days across all groups was consistent as there was no significant difference (*p* > 0.05) between treatment and control (P0) groups. However, further research needs to verify the results because the production data showed similar low egg production (17%–19%), in both control (P0) and at a salinity of 0.75–3.0 PSU. Likewise, there was no effect of water salinity up to 3 gm/l NaCl on the egg quality of Alabio ducks.

## Discussion

Environmental and genetics are the primary factors that strongly influence egg production and quality in laying ducks. Environmental factors include feed, drinking water, temperature, and management, while genetics are hereditary factors inherited from parents, including sexual maturity and the intensity of the interactions between males and females [15]. Studies on salt (NaCl) provision in the ration and drinking water have deteriorated egg production and quality [10,16]. In this current experiment, the production performance (egg production and quality) of Alabio ducks offered freshwater did not differ from those treated with saline water. This result was likely due to insignificant differences in feed and drinking water consumption between treatments, as shown in Table 1. According to Alam et al. [15], every species has an optimum salinity range, and beyond this range, the animal spends more energy on osmoregulation than on other processes.

Feed consumption of the controlled group did not differ from that of the treated groups because the duck’s capability to produce salinity from glandular secretions in their bodies was higher than that from drinking water [17]. This result indicated that Alabio ducks could tolerate NaCl provision up to 3 gm/l by increasing the availability of beneficial bacteria strains, such as probiotics, in the gastrointestinal tract [18]. A study by Iriyanti et al. [19] reported that probiotics in the digestive tract improved the feed intake and production performance of Alabio ducks. Chen and Balnave [16] found no adverse effect of NaCl drinking water on chicken egg production. As a local waterfowl of South Kalimantan, Alabio ducks are adapted to the river environment adjacent to the sea estuary with brackish or salty water that contains high NaCl. Hence, genetics is likely to play a significant role in the salinity tolerance of Alabio ducks.

Ducks are less sensitive to salinity than other waterfowl [17]. This evidence aligns with the current study, which found that Alabio ducks could acclimatize to the saline drinking water up to 3 gm/l without affecting the production performance. However, the difference in the salinity level of drinking water might decrease pathogenic microorganisms in the digestion process. According to Thomas and Wimpenny [20], different levels of NaCl could potentially suppress the growth of pathogenic bacteria, such as Salmonella and Pseudomonas spp., thus improving productivity performance, especially egg production. Zhang et al. [21] stated that the egg production of ducks was exacerbated mainly by the infiltration of pathogenic bacteria such as Salmonella enterica. Hence, the provision of ingredients could reduce pathogenic bacteria to improve the productive performance of laying ducks by optimizing the feed metabolism and thus supporting the availability of required nutrients for egg production [22]. Egg production obtained in this study was substantially lower than that in the experiment carried out by Rostini et al. [4], who reported that the egg production of Alabio ducks ranged between 47% and 81%. The low egg production occurred as the ducks were still in their initial production period (2–3 months). Even though the size of the cages used in this study was right, the ducks did not get used to the system within the first 2 months of production.

**Table 1.** Feed (gm/day) and water intakes (ml/day) of Alabio ducks at different levels of drinking water salinity.

Variables	Treatments^a^				
	P0	P1	P2	P3	P4
Water intake (ml/day)	702.1 ± 9.59	694.8 ± 7.08	703.2 ± 7.50	691.1 ± 8.57	691.2 ± 8.47
Feed intake (gm/day)	128.0 ± 3.45	121.0 ± 1.51	124.3 ± 3.51	124.9 ± 1.43	123.3 ± 3.80

a No significant difference was found (*p* > 0.05).

P0 = control; P1–P4 = treatments (0.75, 1.5, 2.5, and 3.0 PSU).

**Table 2.** Production (%) and quality of Alabio duck eggs (gm) at different levels of drinking water salinity.

Variables	Treatments^a^				
	P0	P1	P2	P3	P4
Egg production (%)	17.3 ± 5.37	18.8 ± 1.93	16.7 ± 3.45	17.3 ± 6.89	17.3 ± 8.45
Egg weight (gm)	61.9 ± 1.68	60.4 ± 2.23	62.7 ± 1.01	63.0 ± 2.34	61.7 ± 2.40
Egg shape index (%)	73.7 ± 0.61	73.9 ± 0.88	74.1 ± 1.77	74.5 ± 1.09	73.3 ± 2.89
Egg density (gm/ml)	1.07 ± 0.03	1.06 ± 0.01	1.06 ± 0.01	1.08 ± 0.01	1.09 ± 0.02
Shell proportion (%)	9.7 ± 0.24	9.9 ± 0.33	9.8 ± 0.45	9.6 ± 0.49	9.6 ± 0.37
Shell thickness (µm)	317 ± 4.76	320 ± 1.63	314 ± 4.24	313 ± 4.97	317 ± 4.86
Yolk index (%)	39.1 ± 1.16	38.2 ± 1.07	36.7 ± 0.98	39.3 ± 1.38	38.0 ± 2.62
Albumen index (%)	3.4 ± 0.40	3.9 ± 0.56	3.4 ± 0.12	3.7 ± 0.11	3.8 ± 0.54
HU	67.0 ± 3.97	70.7 ± 4.59	66.4 ± 1.27	68.7 ± 1.52	70.6 ± 5.82

a No significant difference was found (*p* > 0.05).

P0 = control; P1–P4 = treatments (0.75, 1.5, 2.5, and 3.0 PSU).

In general, there is no significant difference (p > 0.05) in egg quality between treatment and control (P0). Yoselewitz and Balnave [23] stated that NaCl content in the drinking water could deteriorate egg and shell weights due to low water and feed consumption. Although the egg weight found in this study (60–63 gm) was slightly lower than that in previous studies by Rostini et al. [4] (62–67 gm) and by Indarsih et al. [24] (60–65 gm), the weight range was categorized as normal. No adverse effects were identified from the provision of drinking water with a salinity of 0.75–3.0 PSU on the egg shape index, yolk index (%), albumen index, and HU. This finding is consistent with Bennett et al. [17], who pointed out that ducks could adapt to the salinity of drinking water that reaches 3 gm/l without affecting productivity. Likewise, Chen and Balnave [16] revealed that IsaBrown hens could maintain egg production and eggshell quality by including 2 gm/l NaCl in the drinking water. According to Yoselewitz and Balnave [25] and Espinar et al. [26], drinking water with high salinity reduced the shell thickness due to decreased activity of the carbonic anhydrase enzyme in the formation of shells. The shell thickness in the study was in the normal category (313–320 µm) and relatively similar to a study reported by Rostini et al. [4] (309–337 µm). These findings are supported by Alam et al. [15], who noted that poultry that lacks salt gland secretions, such as ruminants, can be affected by high salt levels in the environment.

## Conclusion

The provision of drinking water to Alabio ducks with different salinity levels ranging from 0.75 to 3.0 PSU (equal to 0.75–3.0 gm/l NaCl) did not affect feed and water intakes. Egg production and quality, including weight, shape, density, shell thickness, yolk index, albumen index, and HU of Alabio ducks, were not significantly affected by the increased levels of NaCl in the drinking water. These results showed that Alabio ducks could drink water with up to 3 gm/l PSU of NaCl in it without it affecting their ability to make eggs.

## List of abbreviations

PSU, Practical salinity unit; ME, Metabolisable energy; CP, crude protein; NaCl, Sodium chloride; HU, Haugh unit.
